# Design and Optimization of Nanoporous Materials as Catalysts for Oxygen Evolution Reaction—A Review

**DOI:** 10.3390/molecules29194562

**Published:** 2024-09-25

**Authors:** Zhen Cao, Wenbin Zhang, Tingting Zhou, Wenhui Yan, Kaili Wang

**Affiliations:** College of Chemical Engineering and Environmental Chemistry, Weifang University, Weifang 261061, China; caozhen@wfu.edu.cn (Z.C.); qishisong95460fif@126.com (W.Z.); 15866029951@163.com (W.Y.)

**Keywords:** hydrogen energy, electrochemical water splitting, oxygen evolution reaction, nanoporous materials

## Abstract

With the growing demand for new energy sources, electrochemical water splitting for hydrogen production is a technology that must be vigorously promoted. Therefore, to improve the efficiency of the oxygen evolution reaction (OER) at the anode, high-performance OER catalysts are essential. Given their advantages in electrocatalysis, nanoporous materials have garnered considerable attention in previous studies for OER applications. This review provides a comprehensive overview of various strategies to optimize active site utilization in nanoporous materials. These strategies include regulating pore size and porosity, constructing hierarchical nanoporous structures, and enhancing material conductivity. Additionally, it covers approaches to boost the intrinsic OER activity of nanoporous materials, such as tuning the composition of anions and cations, creating vacancies, constructing interfaces, and forming boundary active sites. While nanoporous materials offer significant potential for advancing OER, challenges remain, including difficulties in quantifying activity within nanopores, the unclear impact of nanoporous material morphology, challenges in accessing nanopore interiors with in situ techniques, and a lack of theoretical calculations on pore structure. However, these challenges also present opportunities, and we hope this review provides a fresh perspective to inspire future research.

## 1. Introduction

With population growth and rising economic levels, global energy consumption demand will double [1,2], necessitating the research and development of new green energy sources to meet the increased demand [3,4,5]. Among conventional energy sources, hydrogen (H_2_) has the highest mass–energy density and can achieve zero pollution emissions [6,7]. Consequently, the efficient utilization of H_2_ energy has become a critical research focus for addressing current energy challenges. Among various H_2_ production technologies, water splitting produces only H_2_ and oxygen (O_2_). In particular, for H_2_ production through electrochemical water splitting, the design of the electrolysis cell allows for the production of H_2_ and O_2_ at the cathode and anode, respectively, directly obtaining high-purity H_2_ and O_2_ [8,9,10,11]. Therefore, electrochemical water splitting for H_2_ production is one of the most promising H_2_ production technologies. Additionally, electrochemical water splitting can generate usable H_2_ cleanly, store surplus electricity from large power grids during off-peak periods, and convert intermittent electricity that is challenging to store sustainably [12].

Electrochemical water splitting encompasses two fundamental half-reactions: the oxygen evolution reaction (OER) occurring at the anode and the hydrogen evolution reaction (HER) taking place at the cathode. The OER, which involves four-electron reactions, requires more stringent reaction conditions and larger overpotential than the HER, posing a substantial barrier to the overall efficiency of the electrochemical water splitting [13,14,15]. Therefore, extensive research has focused on the OER. In addition to coupling with HER for water splitting, OER is an essential reaction in rechargeable metal–air batteries and is crucial for energy storage. Typically, achieving excellent OER performance requires using highly efficient catalysts [5,16,17,18]. Therefore, there is an urgent need to develop effective and stable OER electrocatalysts to promote reactions and improve energy conversion efficiency.

Currently, many highly active OER electrocatalysts have been reported, including precious metal catalysts (such as Ru and Ir) and transition metal catalysts (such as Fe, Co, Ni, and Cu). While the utilization of precious metal catalysts is inherently constrained by their exorbitant costs and exceedingly scarce elemental resources, their unparalleled catalytic prowess continues to evoke immense interest and research endeavors [17,19,20]. Similarly, transition metal-based electrocatalysts are a major focus in alkaline OER research, not only because they are inexpensive and readily available but also because they can exhibit excellent activity comparable with that of noble metal catalysts [21]. It is important to note the wide range of transition metal compounds used in OER, including alloys, hydroxides, perovskites, and more [22,23,24].

For OER catalysts, in addition to selecting the appropriate elements, the structural design of the electrocatalyst is crucial. Enhancing catalyst activity is central to improving catalytic reactions. Recent research on electrocatalysts highlights the need to consider two fundamental parameters: the number of active sites and the intrinsic activity of these sites [25,26]. Both are key factors that influence the overall performance of electrocatalytic reactions. Ideally, the number of active sites and their intrinsic activity should work synergistically, and with optimization, the catalyst’s performance should reach its peak. However, unlike enhancing intrinsic activity, increasing the number of active sites does not linearly improve electrocatalytic performance [26]. This phenomenon can be attributed to several factors, such as the high load on the electrode, which increases the number of active sites, but excessive load hinders electron conduction, thus preventing full performance utilization. Additionally, an excessively thick catalyst layer impedes electrolyte penetration, resulting in an inability to exploit the active sites. These are the key points to consider when designing and improving catalysts, and OER, as a typical electrocatalytic reaction, must follow these principles.

Despite the limitations of increasing the number of active sites on catalysts, this approach remains a popular strategy for improving electrocatalytic performance. The most direct and effective method for increasing active sites is through the construction of nanoporous structures. Nanoporous materials offer several key structural advantages: first, their open pores facilitate electrolyte transport; second, the confined pore space increases the residence time of intermediate species at active sites, promoting the progress of slow reactions. Additionally, by controlling pore size, side reactions can be suppressed, leading to improved selectivity and conversion rates. Lastly, the highly curved inner surfaces of the pores bring multiple active sites into proximity, enabling synergistic performance enhancement [27,28]. Because of their unique advantages in electrocatalysis, nanoporous materials have seen exciting progress in electrochemical energy conversion and storage [29].

The development of nanoporous materials has greatly promoted research into OER, leading to notable recent progress. Although various methods exist for preparing nanoporous materials and constructing nanoporous electrodes—such as template, de-alloying, electrodeposition, and thermal decomposition—the nanoporous materials obtained using any of these methods are endowed with distinct advantages compared with bulk materials and ordinary nanoparticles [29,30,31]. Nanoporous materials not only offer the advantage of exposing more active sites and facilitating electrolyte transport compared to bulk materials, but also, in contrast to ordinary nanomaterials, they possess additional benefits. The unique skeletal structure of nanoporous materials can effectively prevent aggregation during catalytic processes. Moreover, in liquid-phase reactions, nanoporous structures facilitate the removal of bubbles. Another point to mention is that the limiting effect of the pores can turn some slow reactions into dominant reactions [31,32]. These characteristics enhance their performance, and the same benefit applies to using nanoporous materials as electrocatalysts in OER research.

For example, Wu et al. developed high-performance nanoporous electrodes with numerous available surface active sites to reduce the overpotential required for electrocatalytic water splitting reactions [33]. They first prepared mesoporous spinel Co oxide thin films on conductive substrates using dip coating and soft template methods, and the modified material exhibited notable OER performance. Sun et al. also prepared an ordered mesoporous Ni sphere array using the template method. Their research demonstrated the superior OER performance of the material, showing through direct comparison that its electrocatalytic performance outperformed that of ordinary nonporous nanoparticles [34]. The article also mentions that suitable three-dimensional layered porous structures offer excellent structural rigidity and stability in OER. Additionally, Feng et al. developed a universal ligand-assisted self-assembly synthesis strategy [35]. Using the tri-block co-polymer P123 as a template, the metal precursors and carboxyl-containing ligands were coordinated to control the hydrolysis and condensation rates, thereby reducing the cross-linking degree of the metal gel network. This enabled them to prepare high-crystallinity mesoporous transition metal oxides with high surface area and unique stability.

Detsi et al. prepared a high specific surface area mesoporous Ni_60_Fe_30_Mn_10_ alloy/oxide electrode through de-alloying and tested it for OER after proving its excellent porosity [36]. Although they believe that the residual Mn in the electrode might be detrimental to the OER activity, the Ni_60_Fe_30_Mn_10_ alloy–oxide electrode with its porous structure exhibits excellent electrocatalytic performance, requiring only 200 mV of overpotential current density to reach 10 mA cm^−2^ in a 0.5 M potassium hydroxide solution. However, the catalytic ability exhibited by the bulk electrode at this time is negligible. This result highlights the critical importance of exposing numerous active sites in nanoporous materials for OER.

Overall, the introduction of a porous structure enhances the number of catalytic active sites, provides channels for electrolyte transport, and facilitates effective gas removal. Consequently, nanoporous materials outperform bulk materials and nanoporous nanomaterials as electrocatalysts for OER, exhibiting unparalleled advantages. Three-dimensional nanoporous materials can mitigate issues such as agglomeration, catalyst expansion, and fragmentation during electrocatalysis, offering structural stability advantages. On the basis of these factors, nanoporous materials have great potential for OER applications. Given their notable benefits, many researchers are focused on developing novel nanoporous materials to further enhance OER performance, driving progress in new energy science. Numerous researchers have conducted insightful and comprehensive reviews of previous advancements in the application of nanoporous materials for OER. These reviews differ in focus, with some categorizing materials based on synthesis methods—such as thermal decomposition, electrodeposition, templating, and chemical etching—while analyzing the pros and cons of each approach [31]. Others classify nanoporous materials according to catalyst types, including oxides, sulfides, carbides, and phosphides, and thoroughly assess the impact of single metal components and the enhancement of OER performance through element doping [29]. Additionally, some reports explore precursors for the synthesis of nanoporous materials, such as coordination compounds and alloys [30,37]. These comprehensive reviews enable future researchers to quickly understand the application of nanoporous materials in OER and keep pace with relevant development trends, offering valuable guidance for future research.

But what we need to know is that when applied in OER, the key considerations for nanoporous materials are the number of active sites and their intrinsic activity. The number of active sites is typically a natural advantage of nanoporous materials, with further optimization often pursued through morphological aspects. Intrinsic activity encompasses factors such as electronic configuration, defects, and interfaces. Focusing on enhancing the OER performance of nanoporous materials, this review explores two main aspects: the utilization rate of active sites within nanoporous materials and the regulation of intrinsic activity at these sites. To improve the utilization efficiency of active sites, we will examine strategies from three perspectives: increasing the size and optimizing the morphology of pore channels, constructing hierarchical mesopores, and enhancing the conductivity of nanoporous materials. In terms of boosting intrinsic activity, we explore methods such as metal element doping, anion tuning, creating vacancies, constructing interfaces, and forming boundary sites, all of which may contribute to increased activity. Unlike other reviews, this article seeks to provide readers with a fresh perspective on the application of nanoporous materials in the OER field. It aims to explain the underlying mechanisms behind the enhanced OER performance of various nanoporous materials and detail the strategies employed for their optimization. We are confident that by offering insights from diverse perspectives, this review will inspire more comprehensive research, encouraging researchers to optimize nanoporous materials more effectively and promote the synergistic integration of various optimization methods, thereby accelerating progress in OER research.

## 2. Improving the Number and Utilization Rate of Active Sites in Nanoporous Materials

In OER, the enhancement of performance is directly proportional to the number of active sites participating in the reaction. Nevertheless, the emergence of bubbles and the hindrance imposed by liquid surface tension can impede the electrolyte’s access to the interior of nanoporous materials, consequently rendering a portion of the active sites ineffective. Hence, optimizing the utilization efficiency of exposed active sites within nanoporous materials is paramount. Common strategies to regulate the utilization efficiency of active sites in such materials encompass fine-tuning pore size and porosity, constructing hierarchical nanostructures, and enhancing their electrical conductivity. These approaches aim to maximize the accessibility of each active site, thereby bolstering the overall performance of the OER process.

### 2.1. Regulating the Pore Size and Porosity of Nanoporous Materials

**Figure 1:**
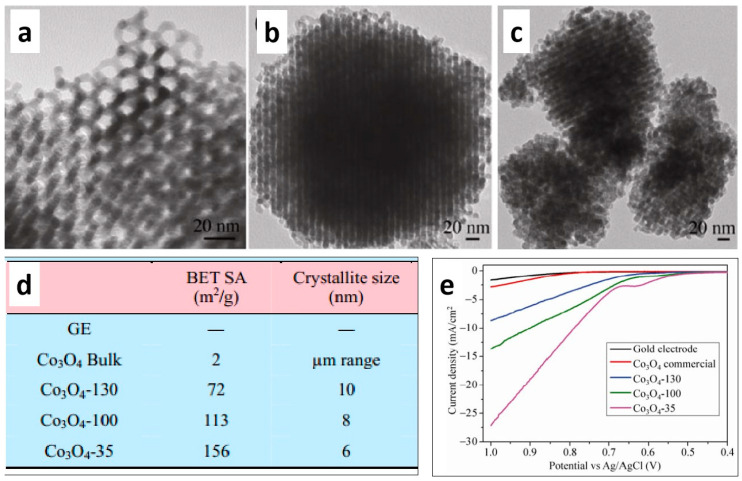
TEM images of (**a**) Co_3_O_4_-139, (**b**) Co_3_O_4_-100, (**c**) Co_3_O_4_-35; (**d**) the BET data and Crystallite size of samples. (**e**) LSV curves of samples. Reprinted with permission from Ref. [38]. Copyright 2012, Tsinghua University Press and Springer-Verlag Berlin Heidelberg.

Nanoporous materials typically have a large specific surface area that can be further increased by regulating their pore size via various methods. A larger surface area means more active sites are exposed, and more active sites allow for improved performance as electrocatalysts for OER. Therefore, Yang et al. synthesized different nanoporous Co_3_O_4_ catalysts using different hard templates to regulate the pore size [38]. Their research showed a clear correlation between increased specific surface area and enhanced OER performance (Figure 1a–d), with Co_3_O_4_ catalysts having the highest surface area and demonstrating the best OER activity (Figure 1e). Further research has also explored the preparation of OER catalysts using the template method, investigating how loading affects the morphology, texture parameters, and other structural aspects during the preparation of porous materials and their effect on OER catalytic performance [39]. In addition to the template method, Chen et al. considered the influence of the number of active sites on the electrocatalytic performance and developed a top-down phase separation dealloying strategy to prepare porous Co_2_P electrodes. This synthesis method offers the advantage of adjustable pore size and porosity, and electrochemical tests have shown that as the pore size decreases, the electrocatalytic performance gradually improves [40]. Guo et al. also demonstrated that mesoporous CoFe-based materials with large specific surface areas exhibit enhanced OER performance [41]. This indicates that porous materials with an increased specific surface area gradually exhibit enhanced electrocatalytic performance as the exposure of active sites increases.

### 2.2. Construction of Hierarchical Nanoporous Materials

From the previous introduction, it is evident that nanoporous materials with small pore sizes, high surface areas, and low densities can serve as efficient catalysts for OER. Although many porous materials have high quality in terms of specific activity, research has found that nanoporous materials with small pore sizes, especially microporous materials, despite having an increased specific surface area, can lead to the incomplete utilization of internal pores, and their electrochemical properties cannot be continuously improved with increasing surface area [42]. This is attributed to the limited mass transfer depth in electrochemical reactions and the blockage of bubbles released by some gas-producing reactions, which hinders effective contact between the electrolyte and active sites [43], resulting in the insufficient utilization of active sites and performance not increasing continuously as the exposed active sites increase. Therefore, if mass transfer can be effectively improved to enhance the utilization of active sites, further performance improvement can be achieved. Typically, increasing the pore diameter improves the electrolyte flux, but this often sacrifices the number of active sites. The construction of graded mesopores has become the most effective solution to this issue because it retains mesopores to maintain the number of active sites while incorporating large pores to address the challenges of limited mass transfer depth and bubble blockage caused by surface tension.

**Figure 2:**
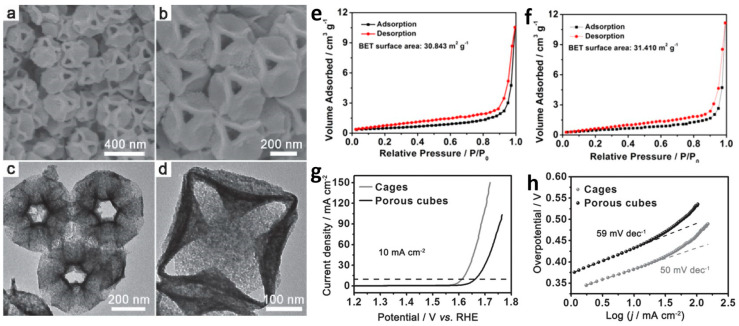
(**a**–**d**) Scanning electron microscopy and transmission electron microscopy images of the CoNi mixed oxide cages. Nitrogen adsorption–desorption isotherms of CoNi oxide (**e**) cages and (**f**) porous cubes. (**g**) Polarization curves and (**h**) Tafel plots of the CoNi mixed oxide cages and porous cubes. Reprinted with permission from Ref. [45]. Copyright 2016, WILEY-VCH Verlag GmbH & Co.

The thermal decomposition of metal coordination polymers, such as metal–organic frameworks (MOFs) and Prussian blue analogs (PBA), is a widely employed method for preparing microporous and mesoporous materials. These metal coordination polymers serve as highly versatile precursors for the development of MOF-derived hierarchical porous materials. Ai et al. quickly thermally decomposed ZIF-67 by regulating the heating rate to obtain a Co_3_O_4_ catalyst with hierarchical pores and demonstrated excellent OER performance [44]. Lou et al. exploited the unsaturated metal coordination and instability of PBA boundaries to selectively etch CoNi PBA nanocubes and obtain hollow CoNi PBA nanoboxes [45]. These nanoboxes were subsequently used as precursors to produce CoNi oxide nanoboxes, which feature both hollow macropores formed by etching of the MOF precursors and micropores–mesopores generated by pyrolysis. The CoNi oxide nanocages demonstrate exceptional OER performance that considerably surpasses that of non-macroporous CoNi oxide nanocubes (Figure 2a–d), highlighting the benefits of hierarchical nanostructures in enhancing the utilization efficiency of active sites. Benefiting from their hierarchical pore structure, nanoboxes exhibit much better OER performance than nanocubes when their specific surface areas are similar. Additionally, Lian et al. used urea solution to selectively etch CoFe PBA under hydrothermal conditions, producing a nanocage [46]. The electrocatalysts that retained the hierarchical pore morphology of the nanocage also exhibited enhanced performance in subsequent electrochemical studies. In addition to employing etching techniques to prepare precursors for hierarchically porous nanomaterials, Lou et al. implemented a directed attachment strategy for growing MOFs, assembling anisotropic nanoparticles into an open framework. This framework is a precursor for a CoFe-based OER electrocatalyst with a hierarchical pore structure. Beyond their use in generating hierarchical porous materials by regulating MOF precursor morphology, the versatility of MOFs as precursors is further exemplified by yielding OER electrocatalysts with hierarchical pore structures. This was achieved by adjusting the heating rate to control the decomposition of the organic ligands [47]. Cao et al. used dealloying to obtain hierarchical nanoporous materials and applied them to the OER [48]. They directly demonstrated that the improved performance of hierarchical nanoporous materials results from the effective utilization of pore channels by comparing the electrochemical surface area (ECSA).

As one of the most widely used methods for preparing porous nanomaterials, the template method is used to construct hierarchical porous materials. Different graded porous materials can be prepared by assembling various soft and hard templates and combining both [49,50]. These hierarchical nanoporous materials exhibit electrochemical performance that surpasses that of single-pore-type materials.

### 2.3. Improving the Conductivity of Nanoporous Materials

**Figure 3:**
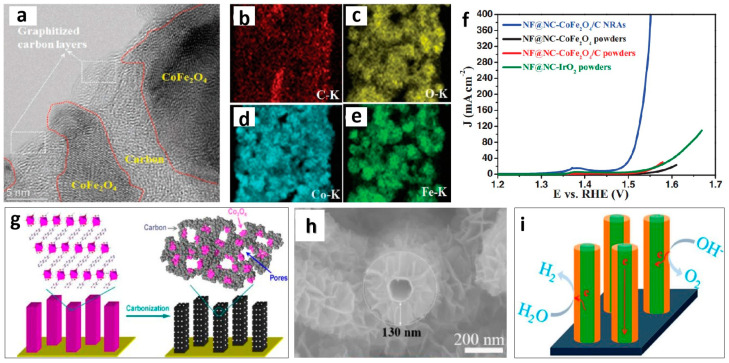
(**a**) High-resolution transmission electron microscopy image of the CoFe_2_O_4_ nanoparticles encapsulated by carbon layers. Elemental mappings of a typical CoFe_2_O_4_@C nanorod, revealing the elemental distributions of (**b**) C, (**c**) O, (**d**) cobalt (Co), and (**e**) iron (Fe). (**f**) Linear sweep voltammetry curves of NF@NC-CoFe_2_O_4_/C nanoarrays, NF@NC-CoFe_2_O_4_ nanopowders, NF@NC-CoFe_2_O_4_/C nanopowders and NF@NC-IrO_2_ nanopowders with the same mass loadings. Reprinted with permission from Ref. [54]. Copyright 2016, WILEY-VCH Verlag GmbH & Co. (**g**) Schematic diagram of preparation of hybrid Co_3_O_4_-carbon nanoporous nanowire array. Reprinted with permission from Ref. [55]. Copyright 2014, American Chemical Society. (**h**) SEM images of CP/CTs/Co-S and (**i**) Schematic of the operating principle of CP/CT/Co-S. Reprinted with permission from Ref. [60]. Copyright 2016, American Chemical Society.

Although many studies have typically attributed conductivity issues to intrinsic activity, in some cases, if the electrolyte effectively contacts the catalyst and electron transfer does not occur at the contact site, the active site cannot be used. Numerous researchers have made substantial efforts to improve the conductivity of nanoporous materials, with carbon materials being highly regarded as excellent conductive carriers. The simplest way to introduce carbon materials is to directly mix them with catalysts to prepare electrodes. However, the conductivity provided by the mechanical mixing of catalysts and conductive carbon materials through ordinary physical contact is limited. An effective method is to directly grow electrocatalysts on conductive carbon materials [51,52,53], with the MOF pyrolysis method gaining notable attention. This method is favored not only for its ease of operation but also because the in situ-generated carbon shell is coated on the surface of the OER active material, effectively protecting the active material from aggregation and thus improving the stability of the catalyst. Li et al. used the MOF pyrolysis carbonization method to prepare a carbon-coated CoFe_2_O_4_ porous nanorod (CoFe_2_O_4_/C) under a high-temperature N_2_ atmosphere (Figure 3a–e) [54]. They also prepared a CoFe_2_O_4_ porous nanorod without carbon shell coating. In comparison, the addition of CoFe_2_O_4_/C with a highly conductive carbon shell exhibited enhanced OER performance (Figure 2f). Additionally, the protection provided by the carbon shell ensured excellent stability. Earlier, Qiao et al. also utilized the MOF decomposition method to synthesize nanoporous materials [55]. They explicitly noted that the carbonaceous material, resulting from the decomposition of MOFs, not only generates nanoporous structures but also anchors active Co_3_O_4_, thereby ensuring electron transfer to the catalytic active sites. Notably, many other noncarbon conductive materials have been introduced into OER catalysis systems to compensate for the insufficient electron transfer in these systems. Similarly, using carbon materials to enhance conductivity, Chen et al. deposited graphene on the surface of nanoporous Ni to improve the OER activity and stability of the catalyst [56]. In addition to carbon materials, other conductive materials are commonly used, such as Ag nanowires [57], Cu_2_O nanorods [58], and nanoporous Au [59].

In addition to catalyst coupling conductive materials, another method of addressing the challenge of electron transfer is to directly grow porous materials on conductive substrates such as conductive glass [61], Ni foam (NF) [62], Cu foam [63], Ti plate [64], and carbon cloth [65]. Zhou et al. used the method of generating a NiFeZn alloy in a NiFe foam and de-alloying it to build a nanoporous structure on the NiFe foam. This electrode exhibits exceptionally high OER performance [66]. Liu et al. also used this method to construct different porous electrodes [23,67,68]. However, when the material layer on the conductive substrate is excessively thick, it cannot ensure electron transfer to catalytic active sites situated at the far end of the substrate. To address this issue, Zhang et al. [60] designed and fabricated an integrated three-dimensional porous electrode composed of carbon paper/carbon tube/Co-S sheets (CP/CT/Co-S). In Figure 3h, Co-S coatings approximately 130 nm thick can be clearly observed on the outer walls of the carbon nanotubes. The conductive carbon tube pores extend from the carbon paper substrate to the outermost edge of the assembled Co-S sheets, ensuring continuous electron transfer from the substrate to the outermost catalytic active sites during electrochemical reactions (Figure 3i). The method of constructing nanoporous materials on conductive substrates uses the conductivity of the substrate and offers the advantage of not having adhesives to cover the active sites. Originating from the effective utilization of increased active sites through electron transfer, this approach of improving the conductivity of porous materials to enhance OER performance is also applicable to other electrochemical technologies as well as key energy conversion and storage technologies.

## 3. Improving the Intrinsic Activity of Nanoporous Materials

### 3.1. Metal Element Doping in Nanoporous Materials

Unlike the utilization efficiency of active sites to enhance the mass transfer efficiency and electron transfer capacity of electrocatalytic processes to increase the maximum current, the enhancement of intrinsic activity can reduce the OER peak potential. Notably, improving intrinsic activity and utilizing active sites are not contradictory and can ideally be solved simultaneously, thereby maximizing electrocatalytic performance.

**Figure 4:**
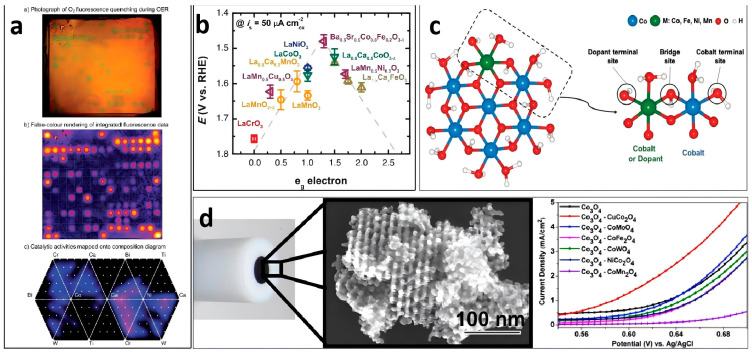
(**a**) Representative photograph of the fluorescence quenching indicative of O_2_ production after 6 min, showing the false-color rendering of the fluorescence data. Activities on the electrode were mapped onto composition diagrams. Reprinted with permission from Ref. [70]. Copyright 2014, The Royal Society of Chemistry. (**b**) Relationship between the oxygen evolution reaction (OER) catalytic activity, defined by the overpotentials at 50 μA cm^−2^, and the occupancy of the e_g_-symmetry electron of the transition metal (B in ABO_3_). Reprinted with permission from Ref. [71]. Copyright 2011, The American Association for the Advancement of Science. (**c**) Pristine or metal-doped cobalt (Co) oxide cluster model with composition Co_6_MO_24_H_27_, highlighting the part of the cluster, including the oxygen sites, considered for the OER mechanism analysis. Reprinted with permission from Ref. [72]. Copyright 2018, WILEY-VCH Verlag GmbH & Co. (**d**) Scanning electron microscopy image of the prepared nanoporous Co_3_O_4_ electrode and linear sweep voltammetry curves. Reprinted with permission from Ref. [73]. Copyright 2013, American Chemical Society.

Typically, the intrinsic activity of surface areas can be fine-tuned by changing the chemical composition of the material. Therefore, bimetallic/multimetal materials provide unprecedented opportunities for electrocatalytic OER, as this operation enhances the surface properties of a catalytically active metal by forming alloys or core–shell structures between two or more metals, thereby changing the intrinsic activity. Bimetallic or multimetal materials have been shown to considerably improve the performance of OER catalysts compared with that of single-metal catalysts [69]. For example, Gerken et al. listed the activity differences of OER in different metal-mixed states through fluorescence quenching (Figure 4a) [70]. Suntivich et al. studied over 10 ABO_3_-type metal compounds and conducted in-depth discussions by introducing molecular orbital theory (Figure 4b) [71]. Furthermore, some researchers have employed theoretical calculation methods to guide experiments, enabling an efficient, rapid, and in-depth understanding of the activity of various metal combinations. For example, using theoretical calculations, Kim et al. demonstrated that the local distortion induced by doping cations can remarkably promote catalysis by adjusting H_2_ bonds at specific active sites (Figure 4c) [72]. For nanoporous materials that expose many active sites, metal doping is an extremely effective method for enhancing their OER activity. For example, Tüysüz et al. prepared a series of metal-doped nanoporous Co_3_O_4_ electrocatalysts by analyzing their linear sweep voltammetry (LSV) curves (Figure 4d). Our observations show that nanoporous Co_3_O_4_ doped with different metals exhibits varying properties [73]. In a recent report, Lu et al. introduced a multicomponent nanoporous (FeCoNi)_2_Nb alloy, which demonstrated excellent OER activity and unparalleled stability as a catalyst [74].

### 3.2. Anion Tuning in Nanoporous Materials

**Figure 5:**
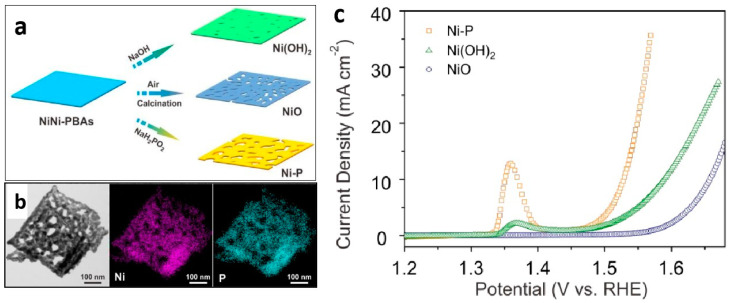
(**a**) Scheme for the formation of Ni(OH)_2_, NiO, and Ni-P porous nanoplates from NiNi-PBAs. (**b**) TEM image and elemental mapping of a single Ni-P porous nanoplate. (**c**) LSV curves of Ni(OH)_2_, NiO, and Ni-P porous nanoplates. Reprinted with permission from Ref. [75]. Copyright 2016, The Royal Society of Chemistry.

In terms of element regulation, changing the anions is an effective strategy for doping metal elements with cations. For instance, Lou et al. successfully prepared nanoporous Ni(OH)_2_, NiO, and Ni–P nanosheets (Figure 5a,b) [75]. In subsequent electrochemical OER tests, the Ni–P porous nanosheets exhibited markedly higher catalytic activity and kinetic performance than Ni(OH)_2_ and NiO porous nanosheets with similar morphologies (Figure 5c). Additionally, some research groups have prepared ordered mesoporous phosphides as OER catalysts using the template method. These phosphide catalysts exhibit excellent performance due to their high specific surface area and improved activity by anion tuning [76]. In addition to metal phosphides, various other anions can improve electrocatalytic OER activity, including sulfides [77], selenides [78], tellurides [79] carbides [80], and nitrides [81]. Notably, tuning both the anions and cations simultaneously can help maximize catalytic activity. Qiao et al. tuned cations and anions while controlling the doping of metal and phosphorus elements in the electrode to prepare electrode materials. Through a comprehensive electrochemical comparison, it has been demonstrated that CoFePO tuned with both anions and cations exhibits superior performance over CoFeOH modified with only cations and CoPO regulated with only anions [82].

### 3.3. Creating Vacancy Defects in Nanoporous Materials

Although simple element doping can rapidly enhance the activity of OER catalysts, it does not provide fundamental insights into the design of such catalysts. Therefore, the further design and construction of material structures are necessary to enhance the activity of catalysts. Many studies have focused on the structural improvement of metal compounds to achieve deeper improvements in catalytic activity. This includes improving the grain size of materials, controlling exposed crystal planes, constructing grain boundaries, and many other methods [83,84,85]. Notably, the creation of vacancy defects on oxide surfaces stands out as a prominent strategy. Several investigations posit that oxygen vacancies (O_v_) play a pivotal role in modulating the electronic structure of catalyst surfaces. They transform cations into a coordination-unsaturated state, thereby facilitating the direct adsorption of H_2_O onto the catalyst’s surface. Additionally, O_v_ act as potent electron donors, potentially shifting the upper boundary of the O 2p band and the Fermi level downwards. This not only reduces the energy gap between the centers of the metal 3d and O 2p bands but also strengthens the covalency of metal-oxygen bonds. Furthermore, the presence of O_v_ significantly amplifies the electronic conductivity of metal oxides, streamlining charge transfer processes and ultimately boosting OER activity. Despite the nuances in the precise mechanisms involved, the consensus remains that oxygen vacancies exert a pronounced enhancing effect on OER performance [86].

**Figure 6:**
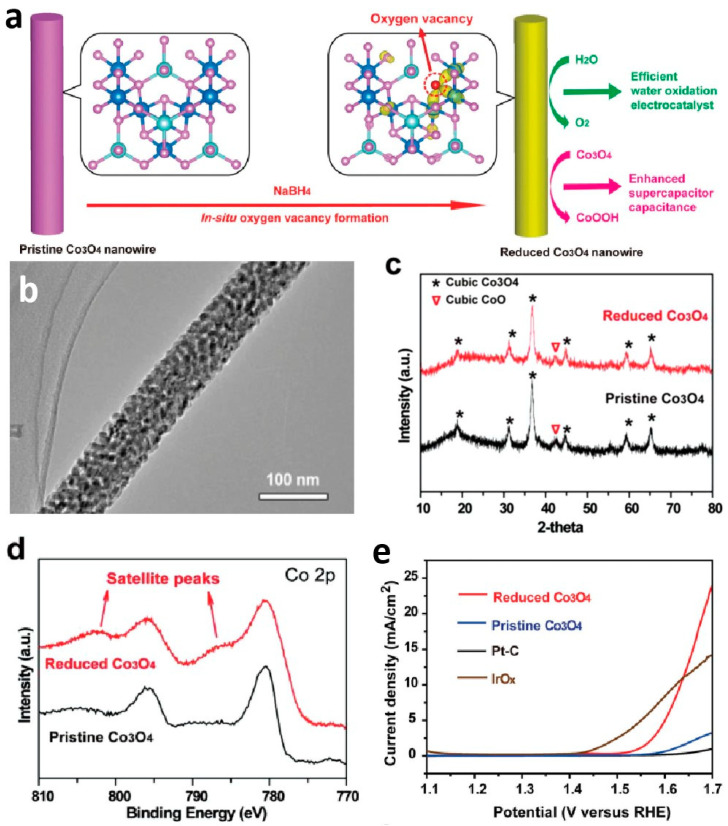
(**a**) Schematic of the NaBH_4_ reduction for the in situ creation of V_O_ in Co_3_O_4_ mesoporous nanowires. (**b**) Transmission electron microscopy image of the reduced Co_3_O_4_ mesoporous nanowires. (**c**) X-ray diffraction patterns, (**d**) X-ray photoelectron spectroscopy data, and (**e**) linear sweep voltammetry curves of the reduced Co_3_O_4_ and the pristine Co_3_O_4_ nanowires. Reprinted with permission from Ref. [87]. Copyright 2014, WILEY-VCH Verlag GmbH & Co.

This approach is crucial for nanoporous materials. Zheng et al. [87] used NaBH_4_ to treat one-dimensional mesoporous Co_3_O_4_ nanowires to obtain rich oxygen vacancy nanoporous materials, further enhancing the electrochemical activity of the exposed active sites by exposing many active sites (Figure 6a,b). To further investigate the surface state of Co, the authors detected the original and reduced Co_3_O_4_ mesoporous nanowires using X-ray photoelectron spectroscopy. Compared with the original Co_3_O_4_ mesoporous nanowires, the reduced Co 2p orbitals show two new satellite peaks located at ~786.2 and ~802.7 eV, indicating the presence of Co^2+^ oxidation states and the reduction of some Co^3+^ ions to Co^2+^ as well as the formation of V_O_ (Figure 6d). After observing the LSV curve (Figure 6e), it is evident that the reduced Co_3_O_4_ mesoporous nanowires exhibit OER activity equivalent to seven times that of the reduced sample under similar morphology and basic structure (Figure 6c). This confirms that the creation of V_O_ effectively improves the intrinsic activity of the material. Additionally, density functional theory calculations confirmed that V_O_ generate new defect states within the bandgap, and the two electrons on the defect can be more easily excited into the conduction band, thereby enhancing the OER activity of Co_3_O_4_. Similarly, Zhou et al. added NaBH_4_ as an auxiliary during de-alloying to obtain a nanoporous Co-based catalyst with increased vacancies. This study also confirms that introducing vacancies enhances the OER activity of porous materials [88].

Additionally, plasma etching can be used to create vacancies and defects. Unlike other defect-processing methods for porous materials, plasma etching simultaneously constructs both porous structures and defects. Dai et al. used this method to directly convert Co_3_O_4_ or NiO from nanosheets into porous and defect-rich materials and intuitively demonstrated through BET normalized current that the improvement in OER performance results from both the increase in specific surface area after creating nanopores and the high activity caused by numerous V_O_ [89].

### 3.4. Building Interface in Nanoporous Materials

**Figure 7:**
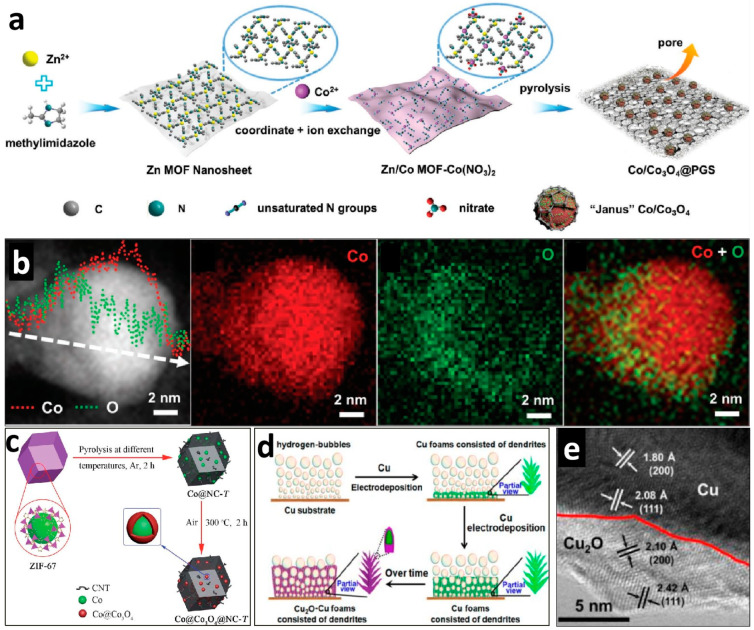
(**a**) Illustration of the synthesis process used to prepare cobalt Co/Co_3_O_4_@PGS. (**b**) Elemental distributions of Co and O in a single Co/Co_3_O_4_@PGS nanoparticle. Reprinted with permission from Ref. [91]. Copyright 2018, WILEY-VCH Verlag GmbH & Co. (**c**) Schematic of the synthetic process of Co@Co_3_O_4_. Reprinted with permission from Ref. [92]. Copyright 2018, The Royal Society of Chemistry. (**d**) Schematic for the fabrication of hybrid Cu_2_O–Cu foams. (**e**) High-resolution transmission electron microscopy image of the interface. Reprinted with permission from Ref. [93]. Copyright 2017, American Chemical Society.

Recently, inspired by the construction of Schottky barriers in photocatalysis to rapidly separate charges, many researchers have used the method of constructing conductor–semiconductor heterojunction interfaces in electrocatalysis to enhance the catalytic activity of materials [90]. Heterojunctions in conductor–semiconductor composite materials often involve phase interfaces that can induce lattice defects. These defects can improve electron transfer efficiency and create additional active sites. Therefore, Chen et al. designed and prepared porous graphitized shell-coated Co/Co_3_O_4_ nanoparticles (Co–Co_3_O_4_@PGS) (Figure 7a,b). This design is expected to enhance the O_2_ catalytic activity [91]. They successfully obtained a product with a Co–Co_3_O_4_ interface by adsorbing Co^2+^ onto ZIF-8 nanosheets and precisely controlling the reduction degree of the Co species using different ratios of Co^2+^ and MOFs. The pyrolysis products of these MOFs possess the porous and active site utilization characteristics of typical MOF-derived materials and the advantage of interface-induced defects that help capture reactive species. Through various effective combinations of porosity and interfaces, Co/Co_3_O_4_@PGS exhibits very high O_2_ catalytic activity. Its activity considerably surpasses that of the comparison samples of elemental Co and Co_3_O_4_ coated with a porous graphitized shell and shows comparable ability with that of precious metal catalysts. In a similar study, Guo et al. directly used ZIF-67 as a Co source and indirectly obtained core–shell structures with Co/Co_3_O_4_ interfaces through heating and oxidation in air, resulting in the formation of the Co@Co_3_O_4_ product (Figure 7c) [92]. This method also produced high-quality electrocatalysts.

Additionally, Li et al. prepared porous materials based on similar considerations using the bubbles generated by cathodic H_2_ evolution during electroreduction as pore templates (Figure 7d) [93]. They constructed nanoporous foams with a Cu_2_O–Cu interface through this regulation (Figure 7e). The hybrid Cu_2_O–Cu porous foam exhibited excellent OER electrocatalytic activity, stability, and reaction kinetics in an alkaline medium. They highlighted that the excellent electrocatalytic performance can be attributed to the unique three-dimensional porous foam, which provides fast transport channels and short-distance diffusion paths for electrolytes, and the core–shell heterogeneous structure, which provides high-speed electron transport and synergistic effects of electrocatalysis.

### 3.5. Creating Boundary Active Sites in Nanoporous Materials

**Figure 8:**
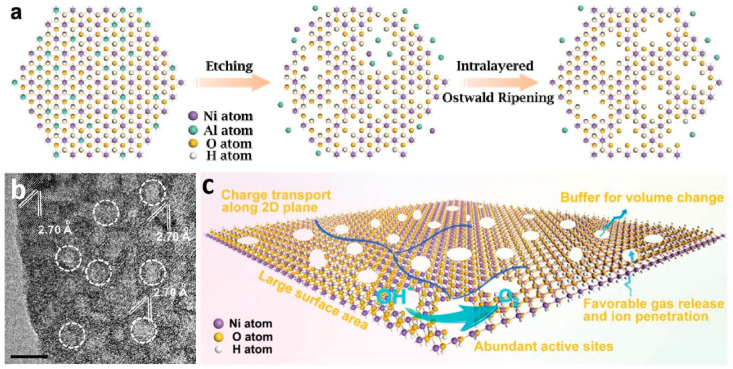
(**a**) Schematic diagram: After Ostwald ripening process, β-Ni(OH)_2_ ultrathin nanomeshes with abundant and uniform nanopores are fabricated from Ni-Al LDH. (**b**) HRTEM image of an β-Ni(OH)_2_ ultrathin nanomesh (Scale bar: 5 nm). (**c**) the structural benefits of the β-Ni(OH)_2_ ultrathin nanomeshes. Reprinted with permission from Ref. [95]. Copyright 2017, WILEY-VCH Verlag GmbH & Co.

Ultrathin two-dimensional materials offer the advantages of high surface atomic exposure and easy charge transfer, and they have received considerable attention in the field of OER. Song and Hu found that when preparing ultrathin layer double hydroxide (LDH) as an OER catalyst using the exfoliation method, OER performance initially increased linearly with increasing ECSA. However, after exfoliation, the activity surged well beyond this linear increase, considerably exceeding the ECSA improvement level [94]. They found that when LDH was exfoliated into a single-layer unit cell, the exposed atoms reached their upper limit. The explosive increase in activity was attributed to LDH fragmentation, which exposed many boundary sites. This behavior is similar to that of amorphous materials with excellent OER catalytic activity. The fundamental reason for the excellent catalytic activity of amorphous materials is the short-range disordered crystal structure, which creates numerous boundary sites in amorphous materials, thereby exhibiting extremely high catalytic activity. Xie et al. took a different approach and obtained uniformly sized β-Ni(OH)_2_ porous ultrathin nanosheets by etching and stripping NiAl LDH ultrathin nanosheets in an alkaline solution and Ostwald ripening (Figure 8a,b) [95]. This type of nanosheet offers advantages that general small-sized nanosheets do not, including exposing many boundary active sites, providing stability owing to its overall structure, facilitating charge transfer in a two-dimensional structure, and effectively transporting ions and gases. On the basis of these advantages, β-Ni(OH)_2_ porous ultrathin nanosheets exhibit extraordinary area- and mass-specific activities (Figure 8c). Notably, unlike the previous method of constructing nanoporous materials and improving OER catalytic activity by changing the composition or structure, this method of creating pores on ultrathin two-dimensional materials actually uses pore channels to impart the material with new properties. LDH obtains boundary sites through etching, essentially forming high-density defects [96]. Similarly, Xie et al. used this method to prepare Fe-doped porous ultrathin NiFe LDH, and its OER catalytic activity was further enhanced [97]. Qiu et al. used CoFeAl LDH supported on oxidized graphene to obtain high-performance OER catalysts after corrosion [98].

### 3.6. Other Methods for Improving the OER Activity of Nanoporous Materials

In addition to several effective methods for improving the catalytic activity of OERs introduced earlier, other crucial methods exist. For example, Zhuang et al. prepared core–shell structure Au@Co_3_O_4_ catalyst, using the electron-withdrawing effect of Au to improve the adsorption capacity of the Co_3_O_4_ surface for O_2_ and enhance the catalytic activity [99]. Ye et al. also used Au nanoclusters capable of effectively improving the OER catalytic activity by generating hot electrons via surface plasmon resonance under illumination conditions [100]. Furthermore, introducing a magnetic field can improve the performance by adjusting the direction of the Lorentz force to remove O_2_ bubbles [101]. Although few reports have discussed introducing effective methods such as the electron-withdrawing effect, hot electrons, light, electricity, and force into porous materials, these methods, combined with the advantages of high active site exposure, effective electrolyte transport, and pore confinement of porous materials to increase the residence time of reactants at active sites, are believed to demonstrate remarkable outstanding catalytic performance.

Additionally, reducing the metal-catalyzed active materials to atomic levels can improve their catalytic activity. Because of the inherent instability of single atoms, they generally need to be fixed onto carriers, with porous carbon materials being the most common [102]. However, coordination fixation on other carriers has been reported. Given that most single-atom catalysts require carriers, their activity is closely related to the coordination environment. Therefore, several studies have focused on exploring and regulating the coordination environment of single-atom catalysts. Regarding the control of the coordination environment, MOFs, which use metals and organic ligands to construct pores, can adjust the coordination environment of metals by controlling the type of ligands. Moreover, metal atoms are relatively independent and can exhibit catalytic activity similar to that of single atoms [103]. As a typical porous material, MOFs are crucial in OER. Because the pores of MOFs are mostly at the microporous level, they face challenges in electrolyte transport and the limited utilization of active sites. However, as a porous material, the previously mentioned methods of improving mass transfer, increasing conductivity, element doping, creating interfaces, and generating defects can improve the OER performance [104,105,106].

## 4. Prospects

Electrochemical H_2_ production is typically limited by the complex four-electron process of anode OER, which requires a high overpotential and severely limits the efficiency of H_2_ production through electrochemical water splitting. As electrocatalysts, nanoporous materials have various advantages, including a low-density porous structure that helps reduce costs, a high specific surface area that maximizes the active site exposure, a three-dimensional cross-linked pore structure that promotes electrolyte transport in electrochemical reactions, local confinement of pores that promote effective reactions and internal bending of pore structures that help different active sites approach and produce synergistic effects conducive to catalysis. These advantages have notably advanced research on nanoporous materials for promoting OER. Therefore, the further development of increasingly efficient nanoporous materials for OERs has become an essential goal for researchers. Recently, researchers have developed various methods to regulate the surface and structural states of nanoporous materials to make them ideal OER catalytic materials. This review explores methods for improving the OER performance of nanoporous materials from two main aspects: regulating the number of active sites and constructing hierarchical porous structures. It also involves improving conductivity, adjusting element composition, and constructing vacancies, interfaces, and boundary sites. Here, to more intuitively observe the progress in research on nanoporous materials in advancing the OER, we summarize the performance indicators of some references mentioned in this review in Table 1.

Currently, substantial progress has been made in researching nanoporous materials for electrocatalytic OER, and many studies have integrated various strategies for regulating these materials to enhance OER performance. However, OER water oxidation is a multistep reaction involving complex proton-coupled electron transfer steps. As a result, nanoporous materials continue to face numerous challenges in advancing OER performance.

Firstly, quantifying the catalytic activity of pore channels in nanoporous materials presents significant challenges. While these materials offer the advantage of exposing a large number of active sites, the pore skeleton creates a relatively complete structure. In contrast, a single nanoparticle can be precisely characterized through monolayer tiling, allowing for accurate quantification of each particle’s activity [107]. The difficulty in assessing pore channel performance also complicates the accurate definition of mechanisms in future research. Therefore, it is essential to conduct further research to more precisely quantify the number of pores within a given range of nanoporous materials in OER studies, rather than relying solely on specific surface area and ECSA as metrics.

Secondly, research on nanoporous materials often focuses on pore size and structure, frequently overlooking the impact of overall morphology on OER performance. Many nanoporous materials are initially synthesized as bulk structures and require ultrasonic treatment to achieve dispersed catalyst suspensions [108,109]. This issue also arises with directly prepared nanopowders. However, the overall morphology of these dispersed nanoporous materials is often neglected, and its influence on OER performance remains an area requiring further investigation.

Thirdly, the complexity of the OER process makes monitoring the reaction pathway challenging, and an accurate reaction mechanism is still under development. This complexity complicates the quantification of the specific efficiency of various methods for improving nanoporous materials. Advanced in situ experiments are needed for real-time detection and tracking [110]. Moreover, nanoporous materials may exhibit different chemical compositions and structures on their surfaces compared to within their pores under OER conditions. This discrepancy can lead to inconsistencies in the mechanisms involved, causing deviations in interpretations.

Fourthly, combining theoretical calculations with experimental analysis can guide subsequent studies to develop nanoporous materials with enhanced OER performance. This approach has the potential to significantly improve the practical application of nanoporous materials in electrochemical water splitting for hydrogen production. However, it is important to acknowledge that theoretical research on the structural models of nanoporous materials remains limited. This includes understanding the impact of various pore structures on stability and the relative merits of ordered versus disordered nanopores.

Despite the challenges faced by nanoporous materials, these obstacles also present opportunities for advancing OER performance. The progress made in this field has significantly improved OER efficiency. We appreciate the numerous researchers dedicated to this field and look forward to developing more efficient and practical electrodes based on nanoporous materials.

## Figures and Tables

**Table 1:** List of the electrocatalytic activities of nanoporous catalysts for OER.

Nanoporous Materials	Electrolyte	Overpotential η_mA cm_^−2^ [mV]	Tafel [mV dec^−1^]	Extended Application Based on OER	Material Groups *	Ref.
meso-Co_3_O_4_ thin film	1 M KOH	η_10_ = 340	57	-	1	[33]
Ordered Mesoporous Ni Sphere Arrays	1 M KOH	η_10_ = 254	39	-	1	[34]
Co_3_O_4_/Fe_3_O_4_(4:1)	1 M KOH	η_10_ = 320	78	-	1	[35]
Mesoporous-Ni_60_Fe_30_Mn_10_	1 M KOH 0.5 M KOH	η_500_ = 360 η_10_ = 200	62 -	-	1	[36]
Co_3_O_4_-35	0.1 M KOH	η_10_ = 525	-	-	2.1	[38]
c-Co_3_O_4_-5	0.1 M KOH 1 M KOH	η_10_ = 496 η_10_ = 410	96 59	-	2.1	[39]
CoFe_2_O_4_@C nanomaterials	1 M KOH	η_10_ = 248	58.7	-	2.1	[41]
Hierarchically porous Co_3_O_4_	0.1 M KOH	η_10_ = 450	89	-	2.2	[44]
CoNi Porous cages	1 M KOH	η_10_ = 380	50	-	2.2	[45]
Co_0_._6_Fe_0_._4_P-1.125	1 M KOH	η_10_ = 298	48	Full water splitting	2.2	[46]
CoFe frame-like superstructure	1 M KOH	η_10_ = 340	57	-	2.2	[47]
HP-CoFe	1 M KOH	η_10_ = 290	49.9	-	2.2	[48]
hierarchical CoOx nanosheet/nanotube	1 M KOH	η_51_._2_ = 420	75	Full water splitting (HER: Pt)	2.2	[50]
NF@NC-CoFe_2_O_4_/C	1 M KOH	η_10_ = 240	45	-	2.3	[54]
Co_3_O_4_C-NA	0.1 M KOH	η_10_ = 290	70	-	2.3	[55]
Ag NW@Co NS	1 M KOH	η_10_ = 320	75.4	-	2.3	[57]
NiFe/Cu_2_O NWs/CF	1 M KOH	η_400_ = 310	42	-	2.3	[58]
nanoporous Au/Cr–NiFe	0.1 M KOH	η_10_ = 323	33	-	2.3	[59]
CP/CTs/Co-S	1 M KOH	η_10_ = 306	72	Full water splitting	2.3	[60]
Ni-NiO/C HPPAs@NF	1 M KOH	η_10_ = 295	52	Full water splitting	2.3	[62]
Ni_2_._2_Fe(OH)*_x_*HNAs	1 M KOH	η_100_ = 298	64.3	-	2.3	[63]
NP CoO-UCSs	0.1 M KOH 1 M KOH	η_10_ = 182 ± 5 η_10_ = 132 ± 4	34	Full water splitting	2.3	[64]
NP-NF@NFF	1 M KOH	η_10_ = 210 η_100_ = 285	32.84	-	2.3	[66]
Cu_x_Co_y_O_4_	0.1 M KOH	η_10_ = 498	-	-	3.1	[73]
NP-(FeCoNi)_2_Nb	1 M KOH	η_10_ = 303	63.6	Anion-exchange-membrane water electrolyzer	3.1	[74]
Ni-P	1 M KOH	η_10_ = 300	64	-	3.2	[75]
Co_3_Ni_1_ P	1 M KOH	η_10_ = 280	66.5	-	3.2	[76]
A-CoS_4_._6_O_0_._6_ PNCs	1 M KOH 0.1 M PBS	η_10_ = 290 η_4_._59_ = 570	67 164	-	3.2	[77]
Ni-Fe-Se cages	1 M KOH	η_10_ = 240 η_100_ = 270	24		3.2	[78]
CoFePO	1 M KOH	η_10_ = 274.5	51.7	Full water splitting	3.2	[82]
Reduced Co_3_O_4_ NWs	1 M KOH	η_13_._1_ = 420	72	-	3.3	[87]
O_V_-Co(OH)_2_	1 M KOH	η_10_ = 350	64.9	-	3.3	[88]
Ar-plasma engraved Co_3_O_4_	1 M KOH	η_10_ = 300	68	-	3.3	[89]
Co/Co_3_O_4_@PGS	0.1 M KOH	η_10_ = 350	52.6	Rechargeable Zn–Air Batteries	3.4	[91]
Co@Co_3_O_4_@NC-900	1 M KOH	η_10_ = 370	94	Rechargeable Zn–Air Batteries	3.4	[92]
Cu_2_O-Cu foams	1 M KOH	η_10_ = 350	67.5	-	3.4	[93]
β-Ni(OH)_2_ ultrathin nanomeshes	1 M KOH	η_20_ = 236	132 (As a Ref.)	-	3.5	[95]
kh-CoNiFe-LDH	1 M KOH	η_10_ = 197	58.3	-	3.5	[96]
NiFe LDH nanomesh	1 M KOH	η_10_ = 268	30	-	3.5	[97]
Fe-CoOOH/G	1 M KOH	η_10_ = 330	37	-	3.5	[98]

* The nanoporous materials mentioned in the table are grouped from different chapters of this review.
